# Interleukin-2/Anti-Interleukin-2 Immune Complex Expands Regulatory T Cells and Reduces Angiotensin II-Induced Aortic Stiffening

**DOI:** 10.1155/2014/126365

**Published:** 2014-09-02

**Authors:** Beenish Majeed, Supannikar Tawinwung, Lance S. Eberson, Timothy W. Secomb, Nicolas Larmonier, Douglas F. Larson

**Affiliations:** ^1^Saver Heart Center, College of Medicine, The University of Arizona, Tucson, AZ 85724, USA; ^2^Department of Pharmacology, College of Medicine, The University of Arizona, Tucson, AZ 85724, USA; ^3^Department of Physiology, College of Medicine, The University of Arizona, Tucson, AZ 85724, USA; ^4^Department of Pediatrics, College of Medicine, The University of Arizona, Tucson, AZ 85724, USA; ^5^Department of Surgery, College of Medicine, The University of Arizona, Tucson, AZ 85724, USA; ^6^Arizona Health Sciences Center, Room 4402, 1501 North Campbell Avenue, Tucson, AZ 85724, USA

## Abstract

Adaptive immune function is implicated in the pathogenesis of vascular disease. Inhibition of T-lymphocyte function has been shown to reduce hypertension, target-organ damage, and vascular stiffness. To study the role of immune inhibitory cells, CD4^+^CD25^+^Foxp3^+^ regulatory T cells (Tregs), on vascular stiffness, we stimulated the proliferation of Treg lymphocytes *in vivo* using a novel cytokine immune complex of Interleukin-2 (IL-2) and anti-IL-2 monoclonal antibody clone JES6-1 (mAb_CD25_). Three-month-old male C57BL/6J mice were treated with IL-2/mAb_CD25_ concomitantly with continuous infusion of angiotensin type 1 receptor agonist, [Val^5^]angiotensin II. Our results indicate that the IL-2/mAb_CD25_ complex effectively induced Treg phenotype expansion by 5-fold in the spleens with minimal effects on total CD4^+^ and CD8^+^ T-lymphocyte numbers. The IL-2/mAb_CD25_ complex inhibited angiotensin II-mediated aortic collagen remodeling and the resulting stiffening, analyzed with *in vivo* pulse wave velocity and effective Young's modulus. Furthermore, the IL-2/mAb_CD25_ complex suppressed angiotensin II-mediated Th17 responses in the lymphoid organs and reduced gene expression of IL-17 as well as T cell and macrophage infiltrates in the aortic tissue. This study provides data that support the protective roles of Tregs in vascular stiffening and highlights the use of the IL-2/mAb_CD25_ complex as a new potential therapy in angiotensin II-related vascular diseases.

## 1. Introduction

Aortic stiffness has been shown to be an independent predictor of cardiovascular disease and mortality in patients with hypertension [1]. It has been shown that aortic stiffness precedes the development of essential hypertension and high initial blood pressure is not always predictive of increased aortic stiffness [2, 3]. Microvascular and endothelial function are impaired or damaged as a direct consequence of aortic stiffness [3]. Moreover, increased pulse pressure associated with arterial stiffness causes end-organ damage, especially in the heart, brain, and the kidneys [4–7]. Since the mechanisms underlying the development of vascular stiffness in large conduit arteries are mostly unknown, we investigated the role of the adaptive immune system in a murine model of angiotensin II- (Ang II-) induced aortic stiffening.

Ang II, the most important component of the renin-angiotensin system, is associated with hypertension and renal failure. Ang II, through the angiotensin II type-1 receptor (AT1-R), is also a potent stimulator of the T-helper- (Th-) 1 and T-helper-17 adaptive immune responses [8–10]. Most notably, angiotensin converting enzyme inhibition promotes regulatory T cell (Treg) expansion [11]. Chronic infusion of an AT1-R agonist results in hypertension and vascular remodeling that is dependent upon the integrity of T cells in the immune system [12, 13] and more specifically the Th17 lymphocyte subset [14]. Therefore, there is a close association among the adaptive immune system, Ang II, and vascular function.

Adoptive transfer of Tregs has shown benefits in Ang II-models of hypertension. In regard to Ang II-induced stiffness of small arteries, it has been shown that adoptive transfer of Tregs reduces stiffness of mesenteric arteries using* ex vivo* analysis of stress versus strain [15]. However, no studies have evaluated the effects of Tregs on the stiffness of large arteries, which is important to consider for their function as elastic reservoirs, a characteristic known as the Windkessel effect. Moreover, adoptive transfer of Tregs requires larger numbers of donors or* in vitro* stimulation and is difficult to translate into the clinical setting.

Recent studies have reported that immune complexes composed of interleukin-2 (IL-2) and the anti-IL-2 monoclonal antibody (mAb) clone JES6-1, abbreviated as mAb_CD25_, because of its ability to direct the binding of IL-2 to CD25-expressing cells, can selectively induce rapid expansion of Tregs with significant immunosuppressive function* in vivo* [16, 17]. These IL-2/mAb_CD25_-expanded Tregs have been shown to prevent mice from experimental autoimmune encephalomyelitis induction [16], suppress collagen-induced arthritis [18], and attenuate the development of atherosclerosis [19]. In this study, we investigated the use of IL-2/mAb_CD25_ to induce Tregs and protect mice from Ang II-induced vascular remodeling and stiffness. Our data demonstrate that IL-2/mAb_CD25_ induces expansion of Treg lymphocytes and prevents Ang II-induced vascular stiffening without lowering blood pressure. These results support the use of IL-2/mAb_CD25_ as a novel therapeutic and have important clinical implications, since arterial stiffening is considered an independent marker for increased cardiovascular diseases.

## 2. Materials and Methods

### 2.1. Animals and Study Design

Male C57BL/6J mice at 3 months of age were obtained from Jackson Laboratories (Bar Harbor, ME, USA). This study was approved by the University of Arizona Animal Care Committee and conformed to the Guide for the Care and Use of Laboratory Animals published by the US National Institutes of Health (NIH Publication number 85-23, revised 1996). All mice were maintained in the animal facility of the University of Arizona and fed with NIH-31 Modified Open Formula Mouse/Rat Sterilizable Diet from Harlan Laboratories and randomly divided into placebo and three treatment groups.

Mice were administered 6 *μ*g per injection of IL-2/mAb_CD25_ (5 *μ*g of anti-IL-2 mAb + 1 *μ*g of IL-2) (R&D Systems) i.p. for 5 consecutive days and 3 times weekly thereafter according to Dinh et al. [19]. It has been shown that this dose, which is equivalent to 1 : 2 molar ratios, gives maximal systemic Treg expansion [16]. After 1 week of IL-2/mAb_CD25_ pretreatment, the AT1-R agonist [Val^5^]angiotensin II (Ang II) (Sigma Chemical Co) was then infused via Alzet osmotic minipumps (model 1004; Alzet, Palo Alto, CA, USA) at a dose of 490 ng/min/kg for 14 days as described by Guzik et al. [12]. The control groups received the pumps filled with phosphate buffered saline (PBS) and/or injections of PBS. All mice were sacrificed after 14 days of Ang II infusion.

### 2.2. Blood Pressure Measurements

All mice were trained with the tail cuff system for 3 consecutive days prior to measurements (Hatteras Instruments, Cary, North Carolina) and data were recorded for days 0, 7, and 14. Tail cuff was also performed for 3 consecutive days before each weekly measurement. Tail cuff measurements were performed with the mice on a heated platform. Blood pressure values were recorded from an average of 10 consecutive measurements with a standard deviation lower than 10.

### 2.3. Perfusion Fixation and Histological Staining

Before perfusion fixation, mice were given a subcutaneous injection of 100 *μ*L of 1,000 USP units/mL Heparin Sodium. Thirty mL of saline was infused to remove vascular blood, followed by the administration of a 2 : 1 solution of 3% glutaraldehyde to 1% formaldehyde at a constant perfusion pressure of 40 mmHg for 15 min to limit fixation contraction. Thoracic aortas were paraffin-embedded, cut in 5 *μ*m sections, and stained with Masson's trichrome for morphometrics and Picrosirius red (PSR) for collagen. Image analysis was performed using NIH ImageJ to quantify aortic wall thickness and collagen content.

### 2.4. Pulse Wave Velocity Measurements of Aortic Stiffness

Noninvasive ECHO Doppler measurements of pulse wave velocity (PWV) were measured with the Vevo 770 using a 45 MHz Doppler probe with a 12.7 mm focal distance (Visual Sonics). Mice were anesthetized with 1.3% isoflurane and imaged in the supine position while secured to heated platform with continuous electrocardiogram (EKG) monitoring. Heart rate was kept between 340 and 360 beats per minute. Due to the explicitly identifiable anatomical locations of the innominate artery and the abdominal aorta at the proximal renal artery bifurcation, these two points were used for defining the transit time difference with respect to the EKG R-wave. The distance between the innominate and renal arteries was measured under direct view with* ex vivo* dissection and divided by the transit time difference to compute the PWV.

### 2.5. Estimation of Wall Mechanical Properties

The Moens-Korteweg equation for pulse wave velocity, *c*, is stated as

(1) $$\begin{array}{c} c={\left(\frac{Eh}{2R\rho }\right)}^{1/2}, \end{array}$$

where *E* is the Young's modulus, *h* is the wall thickness, *R* is the vessel radius, and *ρ* is the density of blood [20]. In order to calculate wall stiffness, *Eh*, from a given pulse wave velocity, the Moens-Korteweg equation was rearranged:

(2) $$\begin{array}{c} Eh=2R\rho c^{2}. \end{array}$$

The Young's modulus, *E*, was then deduced using the data on wall thickness, *h* (including aortic intima, media, and adventitial thickness). Because the arterial wall structure is highly heterogeneous, the values of *Eh* and *E* obtained by this method represent effective values for overall wall properties.

### 2.6. Flow Cytometry

Subsequent to isolation of splenic lymphocytes with Lymphocyte Gradient Separation medium (Mediatech Inc, Herndon, VA), lymphocytes were stimulated with 10 ng/mL PMA, 1 *μ*g/mL ionomycin, and 10 *μ*g/mL Brefeldin A for 5 hours. Surface staining was performed before permeabilization using perm/wash buffer (BD Biosciences, San Diego, CA). Permeabilized cells were subsequently incubated with intracellular-targeting antibodies. Efluor 450-conjugated anti-CD3, FITC-conjugated anti-CD4, Percp-Cy5.5-conjugated anti-CD8a, APC-conjugated anti-CD25, PE-conjugated anti-FOXP3, Percp-Cy5.5-conjugated IL-17, and PE-conjugated IFN-*γ* were purchased from eBioscience. The BD LSR II with BD FACSDiva software flow cytometry system and FlowLogic software (Inivai, VIC, Australia) were used to analyze data.

### 2.7. Semiquantitative Real Time RT-PCR

Aortic tissues harvested from each of the 4 groups were homogenized in TRIzol for RNA extraction (Invitrogen Life Technologies, Carlsbad, CA). Using Rotor-Gene RG-3000 (Corbett Research, San Francisco, CA) the real time PCR was performed with SYBR Green in a 72-well rotor. Using custom designed primers synthesized by Integrated DNA Technologies the following genes were investigated*: F4/80*,* CD3*ε*,* IFN-γ*,* IL-17a*,* procollagen I*α*1*,* procollagen III*,* tropoelastin*, and* prolysyl oxidase*. The gene cycle was normalized by the respective *β*-actin* RNA expression. All data are reported as normalized threshold and fold change of treated groups compared with control.

### 2.8. Statistics

All data are reported as means ± SEM. Comparisons among the defined groups were analyzed by one-way ANOVA, followed by Tukey multiple comparisons tests using GraphPad Prism. For the comparison of treatment groups over time in which mice were paired, repeated measures ANOVA were used with Mauchly's Test of Sphericity, and when this assumption was not met, results were reported based on the Greenhouse-Geisser *F* test. A *P* value of less than 0.05 was considered statistically significant.

## 3. Results

### 3.1. Induction of CD4^+^CD25^+^Foxp3^+^ Lymphocyte Expansion with IL-2/mAb_CD25_ Complex Administration

**Figure 1:**
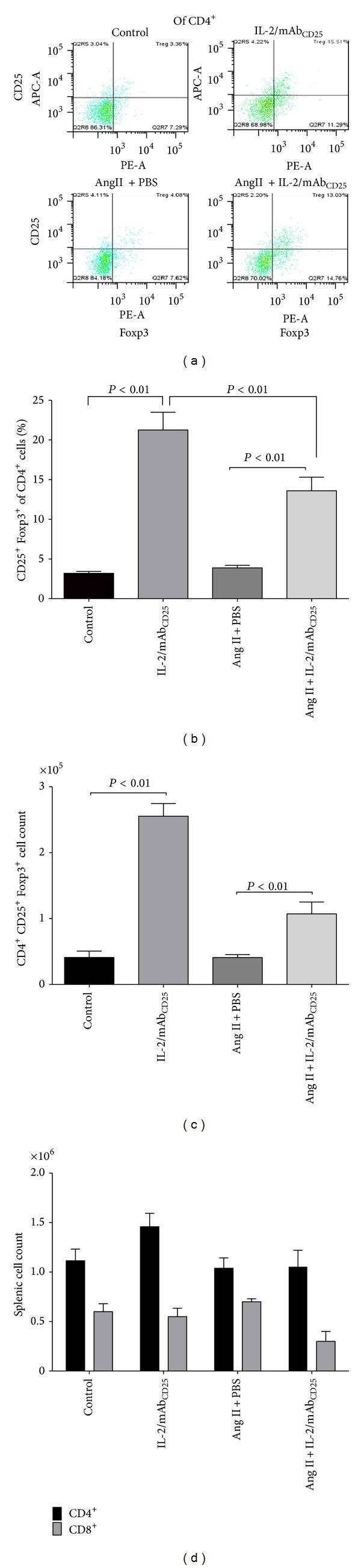
Induction of CD4^+^CD25^+^Foxp3^+^ lymphocyte expansion with the IL-2/mAb_CD25_ complex. Mice were injected i.p. daily with IL-2/anti-IL-2 JES6-1 mAb (IL-2/mAb_CD25_) or PBS for 5 days. Ang II was then infused via osmotic Alzet pumps on day 7. Thereafter, mice received IL-2/anti-IL-2 or PBS 3 times weekly throughout the 14 days of Ang II treatment. Mice receiving PBS infusion serve as a baseline control. Lymphocytes isolated from the spleens were then analyzed with flow cytometry. (a) Representative flow cytometry analysis of Tregs, which express CD4, CD25, and Foxp3. (b) The mean percentage of Tregs gated from the CD4^+^ population shows a significant increase in Tregs after IL-2/mAb_CD25_ complex treatment. (c) Total splenic CD4^+^CD25^+^Foxp3^+^ cell number. (d) Total splenic CD4^+^ and CD8^+^ cell number. *N*  = 7–11 per group. Data are means ± SEM.

Treg expansion induced by IL-2/mAb_CD25_ was verified by flow cytometry. Mice treated with IL-2/mAb_CD25_ or PBS for 5 consecutive days were infused with Ang II or PBS in subcutaneous osmotic pumps starting on Day 7. Thereafter, IL-2/mAb_CD25_ or PBS injections were given 3 times a week. Mice were sacrificed after 14 days of Ang II infusion for flow cytometric analysis of splenocytes. Splenic lymphocytes were gated on the CD4^+^ cell population and analyzed for CD25 and Foxp3 expression.  Figure 1(a) shows representative flow cytometric analysis of splenic Tregs gated on expression of CD25 and Foxp3. Administration of IL-2/mAb_CD25_ led to a 6-fold increase in the percentage of CD4^+^CD25^+^Foxp3^+^ cells in splenic lymphocytes. Ang II infusion alone did not alter the percentage of Tregs. Interestingly, when Ang II was administered with IL-2/mAb_CD25_, the percentage of Tregs increased by 4-fold (*P* < 0.01), which was significantly lower than following treatment with IL-2/mAb_CD25_ alone (Figure 1(b). Total Treg cell numbers were also significantly increased in the spleens of mice treated with IL-2/mAb_CD25_ (*P* < 0.01), and chronic infusion of Ang II diminished the effects of IL-2/mAb_CD25_ on Tregs (Figure 1(c)). Although other studies have shown an increase in aortic CD4^+^ and CD8^+^ infiltrates with Ang II administration [12], splenic CD4^+^ and CD8^+^ cell numbers were not significantly modified by Ang II infusion or by IL-2/mAb_CD25_ complex treatment in this study (Figure 1(d)).

### 3.2. The IL-2/mAb_CD25_ Complex Prevents Vascular Stiffness Induced by Ang II

**Figure 2:**
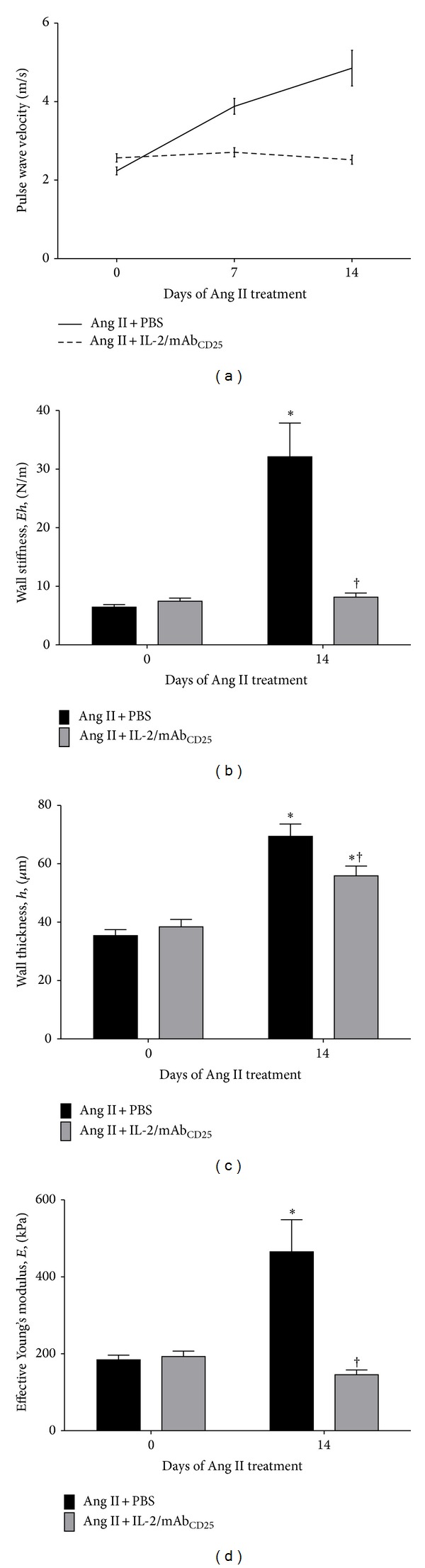
The IL-2/mAb_CD25_ complex prevents vascular stiffness induced by Ang II. (a) Aortic stiffness was determined by* in vivo* measurements of pulse wave velocity with and without immune modulation. (b) Effective wall stiffness (product of Young's modulus and wall thickness, *Eh*) deduced from measured PWV. (c) Wall thickness measured for perfusion fixed aortic specimens that included intimal, medial, and adventitial layers, (*h*). (d) Effective Young's modulus, *E*. Recordings were made weekly during Ang II treatment. *N*  = 5–8. Data are means ± SEM. *P < 0.05 vs Day 0 of respective treatment and ^†^ *P* < 0.05 vs Ang II + PBS at Day 14.

Aortic stiffness was analyzed using pulse wave velocity (PWV) measurements* in vivo*.  Figure 2(a) compares the PWV for treatment using Ang II with IL-2/mAb_CD25_ against Ang II with PBS over 14 days. Initial IL-2/mAb_CD25_ injections did not change baseline values as compared with PBS injected controls at Day 0. Chronic Ang II administration for 14 days led to a time-dependent significant increase in PWV (Greenhouse-Geisser *F* = 27.356, *P* = 0.001). However, stimulation of Tregs with IL-2/mAb_CD25_ administration prevented the Ang II-induced increase in PWV over the 2-week period (groups∗time, Greenhouse-Geisser *F* = 24.005, *P* = 0.001).

Figure 2(b) shows wall stiffness, *Eh*, computed from the measured PWV values. After 14 days, without IL-2/mAb_CD25_, the wall stiffness shows a 5-fold increase that is completely inhibited by the IL-2/mAb_CD25_ complex. The overall wall thickness (intima + media + adventitia) measured with aortic histological specimens harvested caudally and proximal to the renal artery bifurcations is shown in Figure 2(c). After 14 days of Ang II administration, the thickness, *h*, is increased by approximately 2-fold. This increase is only slightly inhibited by the IL-2/mAb_CD25_ complex administration. The effective Young's modulus was estimated by dividing the wall stiffness, *Eh*, by the individual measured wall thickness, *h*, as shown in Figure 2(d). The effective Young's modulus was increased by 2.5-fold, whereas the IL-2/mAb_CD25_ complex completely inhibited the effect of the Ang II-mediated increase in the Young's modulus.

When the Day 14 results are compared with those at Day 0, it is seen that the increase in wall stiffness, *Eh*, results from the combined effects of the increase in Young's modulus and an increase in wall thickness. The reduction in wall stiffness at Day 14 with IL-2/mAb_CD25_ treatment is almost entirely due to the reduction in the effective Young's modulus, with only a slight decrease in wall thickness. The increase in the effective Young's modulus is dependent upon the material composition and/or mechanical properties of the wall rather than the changes in vascular morphometric properties. This implies that the increase in Young's modulus associated with Ang II administration is dependent upon the immune cells that are inhibited by IL-2/mAb_CD25_-stimulated Tregs.

### 3.3. Expansion of Tregs with IL-2/mAb_CD25_ Impairs Ang II-Induced Aortic Remodeling

**Figure 3:**
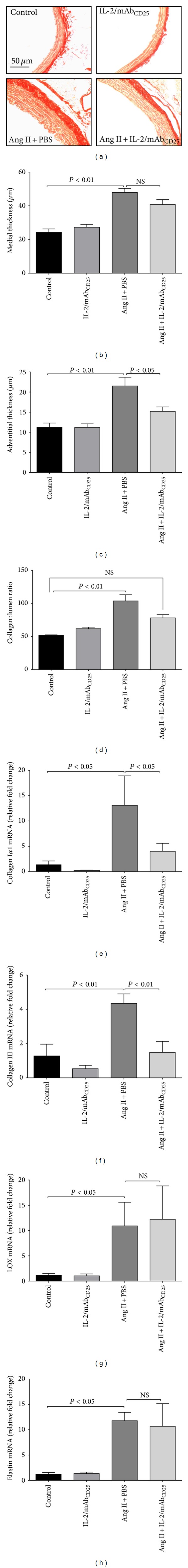
Expansion of Tregs with IL-2/mAb_CD25_ complex impairs Ang II-induced aortic remodeling. Mice were perfusion fixed at Day 14 of Ang II infusion. The lower thoracic aortas were stained with Masson's Trichrome and PSR for histomorphometric analysis. (a) Representative photomicrographs of PSR stained aortic transverse sections from the treatment groups are shown. (b) The mean aortic medial and (c) adventitial thicknesses were quantified. (d) The mean collagen : lumen ratio was measured by PSR intensity. RT-PCR of extracellular matrix genes* procollagen I*α*1* (e),* procollagen III* (f),* tropoelastin* (g), and the cross-linking enzyme* prolysyl oxidase* (h) in the aorta are presented. *N*  = 5–8 per group. Data are means ± SEM.

Immunodeficient mice exhibit blunted vascular hypertrophy in response to Ang II [12] and adoptive transfer of Tregs prevents Ang II-induced vascular dysfunction [15]. We therefore sought to determine whether induction of immunosuppressive Tregs with IL-2/mAb_CD25_ attenuates Ang II-induced aortic remodeling. After 2 weeks of Ang II infusion, the lower thoracic aortas harvested from the control and the treatment groups were stained with Masson's trichrome for evaluating aortic dimension and PSR for collagen distribution and accumulation in the aortas. Representative histological aortic samples from four different groups showed vascular structural alterations in response to Ang II and IL-2/mAb_CD25_ (Figure 3(a)). There was a significant increase in the medial and adventitial thicknesses after 14 days of Ang II infusion only (*P* < 0.01; Figures 3(b) and 3(c)). Coadministration of IL-2/mAb_CD25_ with Ang II significantly reduced adventitial hyperplasia (*P* < 0.05). The increased aortic collagen accumulation with Ang II was prevented with the IL-2/mAb_CD25_ complex (Figure 3(d)). In support of histological remodeling, Ang II-induced mRNA expression of* procollagen I*α*1* and* procollagen III* was significantly reduced by the IL-2/mAb_CD25_ (Figures 3(e) and 3(f)). Gene expression of* tropoelastin* and the cross-linking enzyme* prolysyl oxidase* were not affected by IL-2/mAb_CD25_ (Figures 3(g) and 3(h)). This suggests that although the IL-2/mAb_CD25_ reduces Ang II-mediated collagen gene expression and deposition in the aorta, the effect is selective. This does not exclude the possibility of a reduction in enzymatic activity of lysyl oxidase or changes in quantity elastin protein.

### 3.4. IL-2/mAb_CD25_ Does Not Affect Ang II-Induced Hypertension

**Figure 4:**
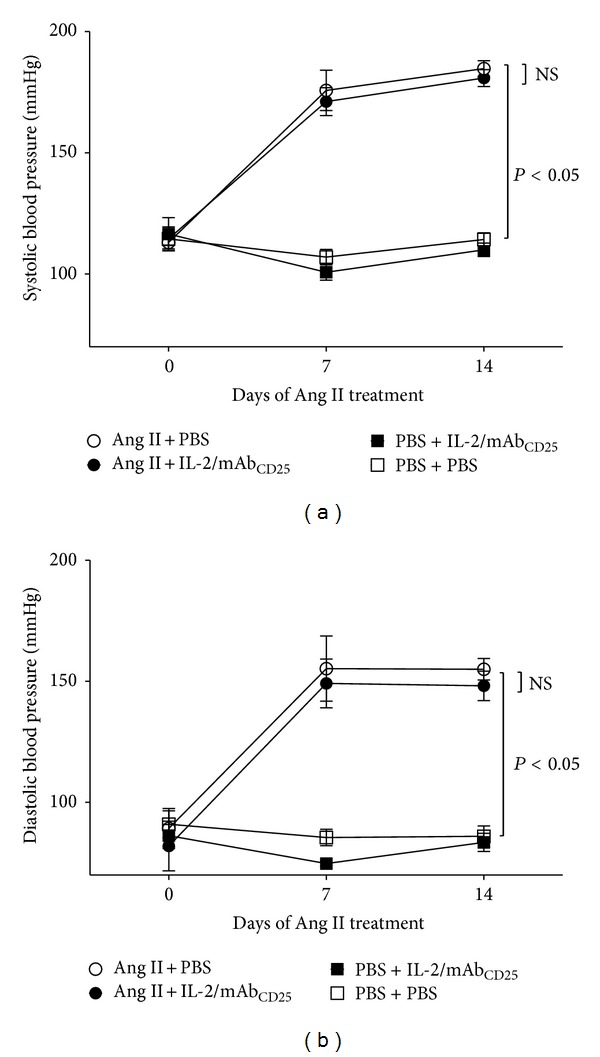
IL-2/mAb_CD25_ does not affect Ang II-induced hypertension. Blood pressure measurements were obtained by tail cuff at Days 0, 7, and 14 of Ang II infusion. The mean values of systolic (a) and diastolic (b) blood pressures in each group are presented. *N* = 8 per group. Data are means ± SEM.

We next determined whether administration of IL-2/mAb_CD25_ complex prevents hypertension in Ang II-infused mice. Using a tail cuff system, systolic and diastolic blood pressure were recorded at baseline and weekly for 2 weeks in trained and acclimated mice. Systolic and diastolic blood pressure in groups treated with Ang II significantly increased over the 14-day period. Treatment with the IL-2/mAb_CD25_ complex did not modify the baseline blood pressure and had no effect on the Ang II-induced increase in systolic or diastolic blood pressure (Figures 4(a) and 4(b)).

### 3.5. IL-2/mAb_CD25_ Administration Inhibits Ang II-Induced Immune Activation in Splenic Tissues

**Figure 5:**
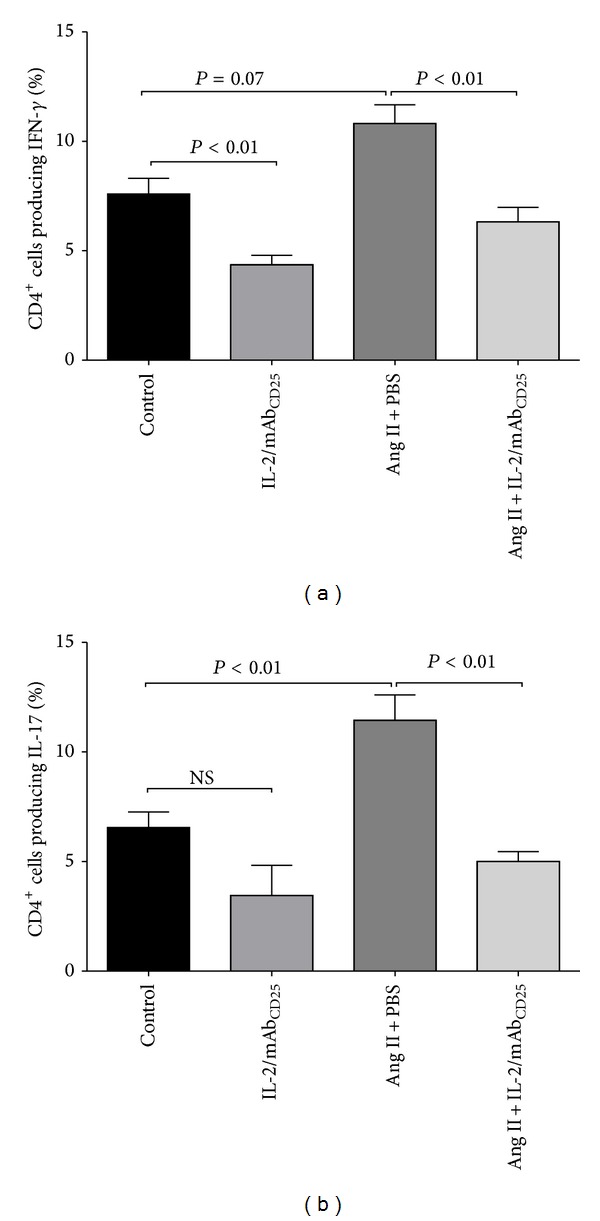
IL-2/mAb_CD25_ administration inhibits Ang II-induced immune activation in splenic tissues. Splenic lymphocytes were isolated and stimulated with PMA, ionomycin, and Brefeldin A and stained for surface markers and intracellular cytokines. The lymphocytes were gated on the CD4^+^ population. The mean percentage of CD4^+^ T cells producing IFN-*γ* (a) and IL-17a (b) is shown. *N* = 8–10 per group. Data are means ± SEM.

Th1 and Th17 lymphocytes participate in Ang II-induced hypertension and target-organ injury [14, 21]. We next sought to further analyze the influence of the IL-2/mAb_CD25_ complex on Ang II-induced Th1 and Th17 responses. Ang II infusion did not significantly increase the percentage of CD4^+^ T cells producing IFN-*γ* (*P* = 0.07; Figure 5(a)). The IL-2/mAb_CD25_ complex significantly reduced the percentage of IFN-*γ* secreting both T cells in the control groups and those induced by Ang II. In addition, splenic CD4^+^ T lymphocytes producing IL-17 were significantly increased with Ang II infusion, which was effectively prevented by IL-2/mAb_CD25_ administration (Figure 5(b)).

### 3.6. IL-2/mAb_CD25_ Suppresses mRNA Expression of Immune Markers in the Aortas

**Figure 6:**
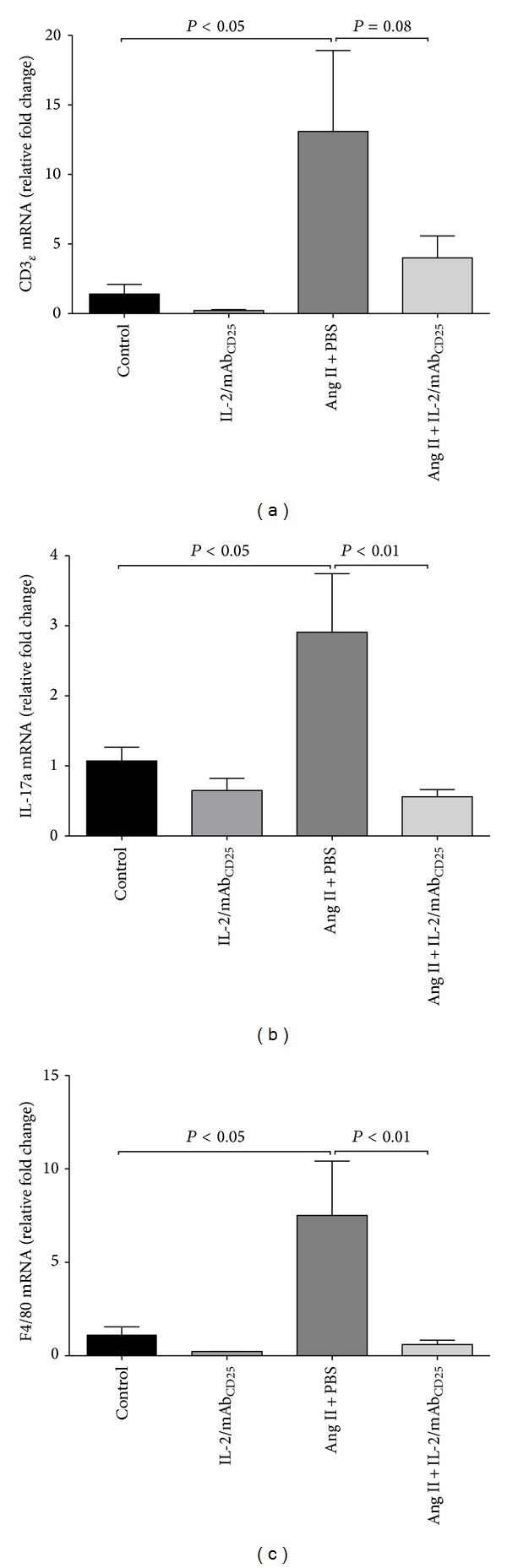
IL-2/mAb_CD25_ suppresses immune cell infiltration and expression of IL-17 in the aortas. Relative gene expression measured by RT-PCR of CD3*ε* (a), IL-17a (b), and F4/80 (c) in the aortic tissues. Gene expression was normalized by *β*-actin expression and reported as relative fold change to control in each panel. *N* = 5–11 per group. Data are means ± SEM.

It has been shown that Ang II induces T cell and macrophage infiltration into the vasculature, which contributes to vascular remodeling [12, 15]. However, immunohistochemical detection of infiltrates was limited in our study (data not shown). We therefore determined the aortic mRNA expression pattern of CD3*ε*, IFN-*γ*, IL-17a, and F4/80 as selective surrogate markers of CD3, Th1, Th17, and lymphocytic and macrophages infiltrates, respectively. Ang II infusion for 14 days led to a 10-fold increase in aortic CD3*ε* gene expression (*P* < 0.05); IL-2/mAb_CD25_ complex administration limited this increase in aortic CD3*ε* mRNA expression (Figure 6(a)). Ang II and the IL-2/mAb_CD25_ immune complex did not change the levels of IFN-*γ* gene expression in the aortic tissues (data not shown). However, IL-17a mRNA expression increased by 3-fold with Ang II infusion (*P* < 0.05), which was prevented with IL-2/mAb_CD25_ immune complex treatment (Figure 6(b)). Ang II infusion also caused a 6-fold increase in aortic F4/80 gene expression, which was abolished by IL-2/mAb_CD25_ treatment (Figure 6(c)). These data therefore suggest that IL-2/mAb_CD25_ suppressed Ang II-immune activation of CD3, Th17, and macrophage infiltrates in the aortic tissues.

## 4. Discussion

The present study was designed to demonstrate the role of the adaptive immune system function in vascular remodeling and stiffening through manipulation of Treg lymphocytes. The immune complex of IL-2 and anti-IL-2 mAb clone, JES6-1 (IL-2/mAb_CD25_), has been shown to induce selective expansion of Tregs with limited or no effects on other cells [17]. The most striking finding of our study was that IL-2/mAb_CD25_ administration completely inhibited the Ang II-dependent increase in the stiffness, quantified with PWV and effective Young's modulus, without affecting the Ang II-induced increase in arterial blood pressure. In summary, this investigation supports the concept that vascular stiffness is dependent on alterations in vascular collagen content, which appears to be linked with proinflammatory Th17 lymphocytes.

Ang II-dependent hypertension and vascular remodeling are associated with immune cell infiltration and T cell activation. In support of previous reports [9, 14], our study demonstrated that infusion of Ang II led to an increase in Th17 lymphocytes in the spleen. Aortic gene expression of the Th17 cytokine, IL-17, was upregulated during Ang II infusion. It is likely that Tregs expanded with the IL-2/mAb_CD25_ complex suppressed proliferation and cytokine production of CD4^+^ T cells producing IL-17. Also, IL-2/mAb_CD25_ treatment reduced the gene expression of aortic T cell and macrophage infiltrates. Further flow cytometric analysis of the aorta could be used to verify these findings.

Since IL-17 is one of the main cytokines released by the Th17 lymphocyte, it follows, therefore, that IL-17 may mediate the vascular remodeling process. IL-17 has been demonstrated to activate fibroblast proliferation via an Akt/miR-101/MKP-1-dependent p38 MAPK and ERK1/2 pathway [22] and fibrosis through the PKC*β*/Erk1/2/NF-*κ*B signaling pathway [23]. It was shown that the fibroblasts constitutively express the IL-17 receptor and IL-17A stimulates fibroblast proliferation and migration [22]. This concept is supported by the observations that IL-17 contributes to liver and pulmonary fibrosis [24, 25]. Furthermore, inhibition of p38 MAPK in particular has been shown* in vitro* to reduce both IL-17A- and stretch-induced collagen expression in aortic fibroblasts and* in vivo* to reduce Ang II-mediated aortic stiffening [26]. Consequently there is support for Th17/IL-17 as a trophic cytokine pathway for the fibroblast induction of fibrosis.

Tregs suppress activation and expansion of multiple innate and adaptive immunocompetent cells and play a critical role in maintaining self-tolerance and immune homeostasis [27]. Recent studies have suggested protective roles of Tregs in hypertension and cardiovascular injury. Adoptive transfer of Tregs reduces cardiac hypertrophy, inflammation, and fibrosis [28, 29], prevents Ang II-induced hypertension and vascular injury [15], and improves coronary arteriolar endothelial dysfunction in Ang II-hypertensive mice [30]. However, the use of Treg adoptive transfer as a therapy may be limited due to low cell numbers present in donor lymphoid organs, requiring large numbers of donors or* in vitro* expansion. Therefore, in this study, we evaluated an alternate and more clinically applicable approach to induce Treg expansion* in vivo* using IL-2/mAb_CD25_ cytokine immune complex therapy as originally described by Boyman et al. [17]. The benefit of this immune complex is that a short treatment period yields a sustained increase in Tregs up to 6-fold. More precisely, the IL-2/mAb_CD25_ complex binds selectively to the relatively small population of CD25-expressing CD4^+^ T lymphocytes and induces* in vivo* expansion the of CD4^+^CD25^+^Foxp3^+^ regulatory T cells [16, 17]. This immune complex, therefore, is an alternative technique that circumvents the limitations of adoptive transfer that include insufficient Treg purity, inadequate numbers for transfer, and potential uncertainty of cell stability. It will be interesting to see the effect of other methods of stimulating Tregs without adoptive transfer, such as inhibition of the chemokine CCL17 [31]. Depletion of CCL17 has been shown to induce Tregs and inhibit atherosclerosis and might also show benefits in Ang II-induced vascular stiffening.

In our study, sustained expansion of Tregs was obtained by repeated injection of IL-2/mAb_CD25_ during the course of Ang II infusion. IL-2/mAb_CD25_ selectively increased Treg numbers in spleens without affecting total CD4^+^ and CD8^+^ T cells numbers. Our results are consistent with previous reports indicating that IL-2/mAb_CD25_ selectively promotes Tregs whereas IL-2 and anti-IL-2 mAb clone S4B6 complexes (IL-2/mAb_CD122_) stimulate proliferation of memory-phenotype CD8^+^ T cells and natural killer cells [32]. Interestingly, although Ang II infusion did not alter the number of splenic Tregs, we found that the percentage of Tregs induced by IL-2/mAb_CD25_ administration was reduced with concomitant Ang II infusion. This observation could be due to Ang II-induction of TNF-*α* [33], which may impair differentiation and function of Tregs through the Akt/Smad3 pathway [34] and dephosphorylation of Foxp3 [35] or possibly by a direct effect of Ang II on the Treg lymphocyte.

Expansion of Tregs with IL-2/mAb_CD25_ administration prevented Ang II-mediated aortic stiffening. Our data suggest that Treg stimulation with IL-2/mAb_CD25_ is effective in inhibiting collagen-dependent aortic stiffening. Inhibition of aortic medial hypertrophy was not observed but aortic collagen accumulation and adventitial thickness were reduced. The tunica adventitia consists of the external elastic lamina, terminal nerve fibers, and surrounding connective tissue, which houses fibroblasts and tissue macrophages. The adventitia has been shown to exert a primary role in the pathogenesis of vascular disease [36] and adventitial remodeling can precede intima and media remodeling [37]. Therefore changes in adventitia may be critical in terms of changes in collagen-mediated aortic stiffness.

Stimulation of Tregs in our study did not prevent hypertension. Some investigators have shown that adoptive transfer of Tregs in Ang II-treated mice prevented or reduced hypertension [15, 29], but Kvakan et al. showed that adoptive transfer of Tregs did not prevent hypertension [28]. We demonstrated that stimulation of Tregs significantly decreases Th1 and Th17 lymphocyte percentages without affecting the hypertensive state. However, a study using IL-17 knockout mice demonstrated a reduced level of Ang II-induced hypertension [14]. These inconsistencies related to blood pressure may be due to differing techniques of Treg augmentation and Treg effects on Th1 and Th17 lymphocyte function and numbers. Our data suggest that, with immunosuppression through Treg induction, Ang II-mediated aortic vascular stiffness is inhibited but the vasoconstrictor activity of Ang II is preserved.

A limitation of this study is that we were not able to detect Foxp3^+^ Tregs in the aortas at a quantifiable level. We cannot presume that the suppression of Th1 and Th17 that we observed after IL-2/mAb_CD25_ treatment is solely due to the ability of the complex to stimulate Tregs without taking into consideration that IL-2 may also directly influence other tissues or cell types. For example, IL-2 signaling through transcription factor STAT5 directly limits IL-17 production and Th17 polarization [38]. In addition, further studies will be necessary to clarify the effect of Tregs in the vasculature during Ang II-induced hypertension. Moreover, we have shown through rtPCR and histomorphometry that collagen content is reduced, which we suggest affects stiffness. However, the contribution of vascular smooth muscle needs to be considered in the stiffness measurements as recently suggested by Saphirstein and Morgan [39].

In summary, our data extend the concept of adaptive immunity in Ang II-dependent hypertension and emphasize a protective role of regulatory T cells in preventing vascular stiffness induced by Ang II. The balance between suppressive regulatory T cells and responder T cells may contribute to a regulation of vascular structure and function in response to Ang II. Stimulation of Tregs with the IL-2/mAb_CD25_ complex could be a new therapeutic approach in Ang II-induced vascular diseases.
